# Supplementary material for: Fund style drift and fund performance: Evidence from China
Source: PLoS One. 2025 Feb 6;20(2):e0316932. doi: 10.1371/journal.pone.0316932 (PMC11801626; doi:10.1371/journal.pone.0316932)
Supplement: Attachment — Submitted filename: Plos 1 Review.docx [file pone.0316932.s001.docx]

Title: Fund Style Drift and Fund Performance: Evidence from China

**Abstract:** The abstract looks too normal. It must be a little innovative to catch the readers' attention. The abstract must include more outcomes from the study, as well as policy implications and recommendations. It must also identify the years, scope, and method applied in the research.

**Introduction**

- The introduction must be reorganized properly. It contains too many smaller paragraphs, which leads to inconsistency and a lack of flow. Most of these paragraphs must be combined.
- The introduction lacks adequate background information as well as statistics to support the idea and readings
- It would be beneficial to clearly state the primary research objectives and focus of your study in the introduction. Consider restructuring your paper to provide a clearer organization of ideas.
- Elaborate on key concepts such as the relationship between the main variables. Provide more detailed explanations and examples to help readers grasp the concepts. Consider integrating recent research to support your research problem and objectives.
- The novelty including the main contributions of the study is missing. The introduction which serves as the bedrock of the study is not what I am expecting to see in a top journal like this.

**Literature**

- This section needs to benefit from a theoretical and empirical literature review to buttress the background of the study. These two sections must be separated in the study
- A literature gap must be developed in the study to capture the differences between the study and previous literature
- I will recommend that a clear hypothesis be developed in this section.
- The authors must end this part by concluding their contribution to the extant literature.

**Data**

- This section must clearly provide some justification for the use of the variables employed in the study.
- More justification is needed on the time span used as well as the countries selected. Avoid stating lack of data as reasons for the selection of time and countries.
- A table must be provided in this section and must include variable definition, data source, years and expected signs
- This section must provide some justification for the use of the variables employed in the study.

**Methods**

- This section must clearly provide some justification for the use of the variables employed in the study.
- More justification is needed on the time span used as well as the countries selected. Avoid stating lack of data as reasons for the selection of time and countries.
- Why did the study dealt with SSA countries?

**Equations and methods**

- A detailed equations and methodological stages must be stated.
- All the methods employed in the study should be grouped under one heading. The importance of these methods must be explained to capture their relevance and their applications.

**Results**

- The discussion of results should emphasize the economic logic and intuition behind the results.
- I will recommend that the author links the discussion with other studies
- Don’t forget to explicitly explain the results.

**Conclusion and Policy Implication**

- In the conclusions section, a good one must be given.
- The implications and recommendations must be related to the results of the study
- More cutting-edge limitations and direction for future studies must be stated
