# Supplementary material for: Fund style drift and fund performance: Evidence from China
Source: PLoS One. 2025 Feb 6;20(2):e0316932. doi: 10.1371/journal.pone.0316932 (PMC11801626; doi:10.1371/journal.pone.0316932)
Supplement: Attachment — Submitted filename: Response to Reviewers.docx [file pone.0316932.s002.docx]

# Reviewer #1 Response

*Reviewer #1:*

*Dear author(s),
I have read your paper and found it very interesting, but still, I have found some major issues and fundamental flaws which needs to be addressed before publication.

1- Abstract: Abstract is the executive summary of all of your paper, however, you have not provided details about methodology and implication.*

**Response**: Thank you for pointing out this issue. We have revised the abstract to include more specific details regarding the methodology and implications. In particular, we describe the bidirectional fixed effects model used for data analysis and introduce a new measurement method for fund style drift based on industry allocation. Additionally, we have elaborated on the practical implications of our findings for fund managers and regulators. We hope these revisions address your concerns comprehensively.

*2- Introduction: In this section you are providing a brief introduction about the issue your investigating in global as well as target context with the help of data, tables, reports, numbers, figures, and justifying your statements by references. In your case, you have mixed your Introduction and Literature review section. It is very difficult to continue the flow and difficult to understand. You have not justified your statements i.e. page 11 para 2 and page 12 para 3,4. Please provided proper justification (citations) for all of your important statements.*

**Response**: Thank you for your valuable feedback. We have carefully revised the introduction, incorporating data, numbers, and reports in the research background to explain better the issues we are investigating. We have provided relevant data on the development scale of actively managed equity funds in China and the prevalence of style drift. Additionally, we have included references to the concept of style drift and its correlation, citing key literature on the subject. Furthermore, we have restructured the introduction to clearly distinguish it from the literature review, addressing your concerns regarding the flow and structure of this section. We now provide a more concise introduction to the research questions, followed by a dedicated literature review that discusses related studies in detail. This reorganization improves the clarity and logical flow of the paper. For the statements in the second paragraph on page 11 and the third and fourth paragraphs on page 12, we have added justifications for the relevant statements (with citations).

*3- Literature Review: It is one of the most important section, in which you are providing what are the "known and unknown" about your topic by providing the latest references to justify your study. In your case, most of the reference are outdated, which shows that nothing has been done in this area or you missed some important information. You have also interpreted some of your results in LR section on page 11 &12.
Please rewrite you LR section and discuss the variables and their relationships with respect to previous studies, provide theoretical support for each of the relationship and come-up with what has been unknown or what needs to be investigated and develop your hypothesis to be tested.*

**Response**: Thank you for your valuable feedback. We have carefully rewritten the literature review section to address the concerns you raised. Specifically, we have made the following revisions:

1. Updated and Expanded the Literature Review: We acknowledge the concern that many of the references in the original version may be outdated. To address this, we have incorporated more recent studies to provide a more comprehensive understanding of the research landscape. These include studies from 2021 and 2024 (Li and Jin, 2021; Koenig and Burghof, 2022; Yi et al., 2024; Liu and Yi, 2024; Zhang and Lv, 2024), which discuss the factors influencing fund style drift and its impact on fund performance. By integrating these newer sources, we aim to reflect the latest developments in the field and offer a clearer understanding of both the known and unknown aspects of the topic.
2. Clarified the Relationships Between Variables: We have discussed each key variable in the study about prior research, providing theoretical support for each relationship and formulating corresponding research hypotheses. We explore both the positive and negative impacts of style drift on fund performance, as discussed in previous studies (e.g., Wermers, 2012; Andreu et al., 2019), and address the reasons behind the conflicting findings in the literature (Huang, 2011; Zhang et al., 2024; Yi et al., 2024). Additionally, in the final paragraph of the literature review, we summarize the relationship between our study and previous research, highlighting areas that warrant further investigation.
3. Identified Knowledge Gaps and Formulated Hypotheses: In response to your comment on addressing the "unknowns" or "gaps" in research, we identified several key gaps in the literature. Specifically, the relationship between industry-style drift and fund performance has not been sufficiently explored, particularly in the context of China. Most existing studies focus on style drift based on market capitalization or growth/value characteristics but do not delve into how industry allocation influences performance. We propose a more targeted measurement of style drift, focusing on industry-level deviations, which have not been adequately addressed in the literature. We also highlight the lack of research on the motivations behind style drift, especially distinguishing between drift driven by investment ability and trend-chasing drift, and how these two types of drift differentially impact performance.
4. Revised Hypotheses: Based on the latest literature and identified gaps, we have added and clarified our hypotheses. We now explicitly test the relationship between different types of style drift and fund performance.

*4- Methodology: In this section you are discussing your sampling procedure, variable measurement, estimation model, hypothesis testing and justifying each one by providing references.*

**Response**: Thank you for your feedback. We have revised the methodology section to provide clearer explanations and justifications for each part. Below are the key changes we made:

1. We have specified the data sources from the WIND and RESSET databases and explained the rationale for selecting Chinese open-end funds from 2007 to 2022 as the research sample. The Chinese fund market is highly influenced by policies, and regulatory bodies such as the China Securities Regulatory Commission (CSRC) have implemented a series of policy documents to limit style drift and regulate market behavior. This policy intervention provides a unique context for studying fund style drift and its impact. The decision to start the study in 2007 was made for two reasons: first, the number of Chinese open-end equity funds was relatively small before 2007, and second, fund information disclosure was incomplete before this time.
2. We have also added references for each variable used in the variable measurement section, which can be found on page 10. Furthermore, we have clarified that the empirical model includes both fund-fixed effects and time-fixed effects to account for unobserved heterogeneity.

*5- Findings/Results: On page 17, Figure1 Panel B, at the bottom of the table, you mentioned that "t-statistics is in parenthesis" however, there is no parenthesis in the table. Panel B of Table 1, most of the interpretation and of the significance are wrong, i.e. CAMP alpha, Size, Fundage etc.*

**Response**: Thank you for your observation. We have corrected the labeling at the bottom of Table 1 regarding the “t-statistics in parentheses” to ensure that the table labels are consistent with the actual table content. Additionally, we have revised the interpretation of the results, particularly regarding the significance of CAPM alpha, Size, Fund Age, and other variables. The difference between the average original excess return and CAPM alpha is significant at the 1% level, and the difference between three-factor alpha and five-factor alpha is significant at the 5% level.

*6- Discussion: In this section, you have to compare your result with previous studies and provided the justification for the consistency or differences. In your paper, you have not provided the discussion.*

**Response**: Thank you for your feedback. We have added a discussion section to address your comments. In the updated version, we compare our findings with previous research in Chapter 8 and provide explanations for observed consistencies or discrepancies, as well as outline the limitations of our study and potential directions for future research. Specifically:

1. Our study differs from previous literature in several ways. First, we examine style drift from an industry allocation perspective, whereas previous studies often focused on fundamental characteristics such as stock size or book-to-market ratios. Second, we measure style drift using benchmark industry allocation differences, which allow for cross-fund comparisons and help identify whether a fund deviates from its investment objective. Furthermore, our use of quarterly data enables a more timely reflection of style adjustments, whereas previous studies predominantly relied on annual data.
2. Additionally, we further explore the behavioral differences in style drift, distinguishing between investment ability-driven drift and trend-chasing drift. Our findings suggest that fund managers with strong stock-picking or market-timing abilities can enhance fund performance through style drift while trend-chasing drift tends to lead to performance deterioration due to increased transaction costs and decision risks. By analyzing the effects of different types of style drift, this study not only explains the discrepancies in existing research but also reveals the complexity of style drift's impact on performance.

However, there are still some limitations in this study. Future research could further improve the measurement of style drift. Although we measure style drift by changes in industry allocation proportions, which effectively capture the intensity and frequency of drift, we have not precisely analyzed the direction of the drift. Future studies could incorporate measures of drift direction to more accurately assess the impact of industry performance on funds. Moreover, this study does not explore the transmission mechanism of style drift within fund networks or its broader market impact. The fund market is a highly interconnected network, and future research could use complex network analysis to investigate how style drift spreads across funds and its potential impact on the overall market, thereby providing regulators with more targeted early warning mechanisms.

*7- Writing: You have to proofread your paper paper as i have noted number of grammatical mistakes.*

**Response**: Thank you for pointing this out. We have carefully proofread the entire paper and corrected the identified grammatical errors.

*Overall; The overall papers is looks like a copy paste from a thesis. You have to re-write the paper with proper format; includes Introduction, LR, Methodology, Results/Findings, Discussion and Conclusion. Thank you*

**Response**: Thank you for your valuable feedback. We have carefully revised the structure of the paper to ensure it adheres to the correct academic format. The paper is now divided into distinct sections: Introduction, Literature Review, Methodology, Results/Findings, Discussion, and Conclusion, in line with standard academic structure. We have also ensured that each section is written coherently, maintaining a logical flow from one section to the next, with appropriate transitions and a more formal academic tone.

# Reviewer #2 Response

*Reviewer #2: Title: Fund Style Drift and Fund Performance: Evidence from China*

*Abstract: The abstract looks too normal. It must be a little innovative to catch the readers' attention. The abstract must include more outcomes from the study, as well as policy implications and recommendations. It must also identify the years, scope, and method applied in the research.*

*Introduction
• The introduction must be reorganized properly. It contains too many smaller paragraphs, which leads to inconsistency and a lack of flow. Most of these paragraphs must be combined.*

**Response**: Thank you for your constructive feedback. We have made the following revisions based on your suggestions:

In the abstract, we introduced the new methodology used in this paper to make it more engaging and informative. We also highlighted the key findings of the study, such as the relationship between fund style drift and fund performance, along with policy recommendations for fund managers and regulators. Additionally, we clarified the research years and scope of the sample used in the study, and elaborated on the methodology, including the regression model based on industry allocation drift and various risk-adjusted return models. We have reorganized the introduction to improve its flow and coherence. Smaller paragraphs have been merged to create a more cohesive narrative.

*• The introduction lacks adequate background information as well as statistics to support the idea and readings*

**Response**: Thank you for your valuable comments. We have revised the introduction to include more comprehensive background information and supporting statistics. Specifically, we have added relevant data to highlight the growth and significance of the Chinese fund market (e.g., the number of actively managed equity funds and total assets). We also incorporated previous research and statistical data to emphasize the prevalence of the style drift phenomenon. These additions provide a stronger foundation for the study and better support the research questions and objectives.

*• It would be beneficial to clearly state the primary research objectives and focus of your study in the introduction. Consider restructuring your paper to provide a clearer organization of ideas.*

**Response**: Thank you for your valuable suggestions. We have revised the introduction to clearly state the main research objectives and focus. The updated introduction now provides a clear overview of the purpose of studying fund style drift and its impact on fund performance within the context of the Chinese market. Additionally, we have reorganized the introduction to improve the organization of ideas, ensuring a clearer flow from the background to the research questions and objectives.

*• Elaborate on key concepts such as the relationship between the main variables. Provide more detailed explanations and examples to help readers grasp the concepts. Consider integrating recent research to support your research problem and objectives.*

**Response**: Thank you for your insightful comments. We have revised the introduction to provide a more detailed explanation of key concepts, particularly the relationship between main variables such as fund style drift and fund performance. We now explain in detail how style drift can either enhance or diminish fund performance, depending on underlying motivations and market conditions. Additionally, we have provided examples to illustrate these concepts, such as how funds that strategically adjust their investment styles based on changing market conditions may outperform those that strictly adhere to their original strategies. We have also included the latest research findings (Li and Jin, 2021; Koenig and Burghof, 2022; Yi et al., 2024; Liu and Yi, 2024; Zhang and Lv, 2024) to strengthen the theoretical background and support the research questions and objectives.

• *The novelty including the main contributions of the study is missing. The introduction which serves as the bedrock of the study is not what I am expecting to see in a top journal like this.*

**Response**: Thank you for your feedback. We have restructured the innovation points and main contributions of the paper. The specific content is as follows:

This paper may make contributions in the following aspects. Firstly, while prior literature focuses on the overall impact of fund style drift, this study differentiates the effects of distinct types of fund style drift behaviors on fund performance. This study explores the impact of style drift based on fund investment ability and trend-chasing style drift on performance, offering new insights into the economic consequences of style drift controversies. Secondly, past research often categorized fund styles based on market capitalization (large, medium, small) and growth/value characteristics (Chan et al., 2002; Idzorek and Bertsch, 2004). In contrast, this study emphasizes industry classification, as investors frequently obtain information about a fund's industry classification. The study offers an approach for quantifying style drift in industry-themed funds. Lastly, the study of the impact of style drift on fund performance not only enriches the literature on the economic consequences of style drift, but also examines whether style drift protects the interests of investors, deepens our understanding of the value of active management of funds, guides investors to rationally analyze popular funds in the market, and treat fund style drift with caution.

*Literature
• This section needs to benefit from a theoretical and empirical literature review to buttress the background of the study. These two sections must be separated in the study*

**Response**: Thank you for your valuable comments. We have revised the literature review section, separating it from the introduction. We now provide a detailed discussion of both the theoretical and empirical literature. We have elaborated on the theoretical foundations of fund style drift, explaining the key concepts and theories behind the study, including an exploration of active management theory and the role of style drift in portfolio construction. Additionally, we have referenced various empirical studies that explore different methods and findings, which provide a stronger background for our research hypotheses.

*• A literature gap must be developed in the study to capture the differences between the study and previous literature*

**Response**: Thank you for your constructive feedback. We have revised the literature review section to clearly highlight the gaps in the literature and the unique contributions of our study. Specifically, we identified the following gaps:

1. Focus on industry classification. While most previous studies use market capitalization or growth/value characteristics to measure style drift, this study introduces a new approach by focusing on industry classification, which better reflects real-world investor information and decision-making.
2. Distinction of style drift motivations. Existing research rarely distinguishes between style drift driven by investment ability and style drift driven by trend-chasing, leading to inconsistent conclusions about the impact of style drift on fund performance. Our study addresses this issue by analyzing these two types of style drift separately.
3. Measurement frequency and scope. Unlike previous studies that often rely on annual data, this study uses quarterly data to capture more timely adjustments in fund strategies, providing a dynamic perspective on style drift.

*• I will recommend that a clear hypothesis be developed in this section.*

**Response**: Thank you for your suggestions. We have revised the literature review section and clearly stated the hypotheses of this study. Specifically, based on theoretical and empirical literature, we propose the following hypotheses:

Hypothesis 1: The stronger the degree of fund style drift, the better the fund performance.

Hypothesis 2: Funds with higher investment ability will achieve higher excess returns when engaging in style drift.

Hypothesis 3: The excess returns of funds engaging in style drift based on market trends will be reduced.

• *The authors must end this part by concluding their contribution to the extant literature.*

**Response**: Thank you for your valuable suggestions. At the end of the literature review section, we summarize the contributions of this study to the existing literature. Specifically, by reviewing both domestic and international research, we find that research on fund style drift at the industry classification level is still in its early stages, lacking a systematic analytical framework and measurement methods. In particular, research on the impact mechanisms of industry dynamic adjustments on style drift is relatively weak. Existing literature mostly focuses on measuring style drift based on market capitalization or style categories, neglecting the dynamic changes in industry allocation and their impact on fund performance. Furthermore, regarding the impact of style drift on fund performance, there is significant disagreement in the existing research, and the heterogeneity of fund managers' motivations has not been adequately considered.

To address these gaps, this study innovatively proposes a method for measuring style drift based on the industry allocation ratios of funds. By quantifying the deviation between a fund's holdings and the benchmark industry allocation, this method provides a more precise reflection of changes in fund style. Furthermore, we categorize style drift into two types: investment ability-driven and trend-chasing-driven style drift. By analyzing the heterogeneous effects of these two types, we not only explain the discrepancies in the literature but also reveal the complexity of style drift's impact on fund performance, providing new theoretical perspectives and empirical evidence for the field.

*Data
• This section must clearly provide some justification for the use of the variables employed in the study.*

**Response**: Thank you for your valuable suggestions. We have modified the data and methodology section to justify the use of each variable in the study. Specifically:

1. Dependent Variable (Fund Performance): We have selected multiple risk-adjusted fund performance indicators to comprehensively evaluate the performance of different models. These indicators are widely used in the literature to measure market risk and are relevant to the Chinese market environment (Fama and French, 1993; 2014; 2016).
2. Independent Variable (Fund Style Drift): Fund style drift is measured by the Euclidean distance between the fund portfolio and its benchmark, reflecting the deviation in industry allocation. This method is based on prior research (Buncic et al., 2015) and is particularly suitable for analyzing style drift in specific industries.
3. Control Variables: We have added the relevant references for the selection and calculation methods of each control variable, as detailed on page 10.

*• More justification is needed on the time span used as well as the countries selected. Avoid stating lack of data as reasons for the selection of time and countries.*

**Response**: Thank you for your comments. We have revised the data section to provide stronger justification for selecting the period from 2007 to 2022 and for focusing on China as the research context. Specifically, the Chinese fund market is heavily influenced by policies, with regulators such as the China Securities Regulatory Commission (CSRC) issuing a series of policy documents to restrict style drift and regulate market behavior. This policy intervention provides a unique context for studying fund style drift and its impact. The choice of 2007 as the starting point is due to the relatively small number of open-end equity funds in China before that year, as well as incomplete fund disclosure before 2007.

*• A table must be provided in this section and must include variable definition, data source, years and expected signs*

**Response**: Thank you for your suggestion. We have revised the Data section to include a comprehensive table in Table 1 that provides the details for each variable.

*• This section must provide some justification for the use of the variables employed in the study.*

**Response**: Thank you for your comments. We have revised the data section to provide a clear justification for the variables used: we use risk-adjusted indicators such as CAPM alpha and the Fama-French model, which are standard for evaluating fund performance. Fund style drift (the explanatory variable) is measured using the Euclidean distance between the fund portfolio and its benchmark, capturing deviations in industry allocation according to established methods. We have also included variables such as fund size, age, and fund manager characteristics, based on prior research, to account for other factors affecting performance.

*Methods
• This section must clearly provide some justification for the use of the variables employed in the study.*

**Response**: Thank you for your feedback. We have revised the "Methodology" section to clearly explain the reasons behind the selection of the variables used and the sources referenced to explain the factors affecting fund performance, as detailed on page 10.

*• More justification is needed on the time span used as well as the countries selected. Avoid stating lack of data as reasons for the selection of time and countries.*

**Response**: Thank you for your comments. We have revised the methodology section to provide stronger justification for the choice of period and country. Specifically, the Chinese fund market is highly influenced by policy, with regulatory bodies such as the China Securities Regulatory Commission (CSRC) issuing a series of policy documents to restrict style drift and regulate market behavior. This policy intervention provides a unique context for studying fund style drift and its effects. The choice of 2007 as the starting point for the study is due to two reasons: first, before 2007, the number of open-ended equity funds in China was relatively small; second, fund information disclosure was inadequate before 2007.

*• Why did the study dealt with SSA countries?*

**Response**: Thank you for your comments. Our study does not focus on SSA (Sub-Saharan African) countries. Instead, it examines the mutual fund industry in China from 2007 to 2022.

*Equations and methods
• A detailed equations and methodological stages must be stated.*

**Response**: Thank you for your suggestion. We have revised the methodology section to provide a more detailed explanation of the equations and stages used in the study. Specifically, we present the regression models used to analyze the relationship between fund style drift and fund performance. We also elaborate on the methodological steps, including data preprocessing, such as winsorizing continuous variables to handle outliers. The style drift is measured using the Euclidean distance between the fund's portfolio and its benchmark. We control for fund characteristics (e.g., size, age) and manager characteristics (e.g., tenure, education) to account for other factors affecting performance.

*• All the methods employed in the study should be grouped under one heading. The importance of these methods must be explained to capture their relevance and their applications.*

**Response**: Thank you for your helpful suggestion. We have revised the "Methodology" section by categorizing all the methods under one heading to improve clarity and organization. Additionally, we have explained the importance of each method and its relevance to the study. The double fixed-effects regression model helps control for unobservable factors that might affect the experimental results. Using the Euclidean distance to measure style drift is crucial for analyzing industry allocation biases and their impact on fund performance.

*Results
• The discussion of results should emphasize the economic logic and intuition behind the results.*

**Response**: Thank you for your suggestion. We have revised the results section to place greater emphasis on the economic logic and intuition behind the findings. Specifically, we now explain how the results reflect the relationship between style drift and fund performance, highlighting why funds with greater style drift may perform better due to active management of market opportunities. We also discuss the impact of fund manager ability, explaining that the performance improvement of style-drifting funds often benefits from the investment skills of the fund managers, while funds driven by trend-chasing behavior may perform poorly due to increased risks and costs.

*• I will recommend that the author links the discussion with other studies*

**Response**: Thank you for your valuable suggestion. We have revised the discussion section to better link our findings with previous research. Specifically, we compared our results with existing literature on fund style drift (e.g., Avramov & Wermers, 2005; Andreu et al., 2019) to highlight similarities or differences between our findings and prior studies. We also discussed our methodology, particularly how focusing on industry classification and distinguishing between investment ability-driven drift and trend-chasing drift expands the current body of work, providing new insights into the economic consequences of style drift.

*• Don’t forget to explicitly explain the results.*

**Response**: Thank you for your feedback. We have revised the results section to more clearly explain the findings. We now explicitly explain the relationship between fund style drift and fund performance, detailing how greater style drift driven by investment ability is associated with performance improvements while trend-chasing drift leads to underperformance due to higher risks and transaction costs. Each key result is discussed within the context of economic intuition and aligned with the study's hypotheses, ensuring that the explanations are clear and directly related to the research findings.

*Conclusion and Policy Implication
• In the conclusions section, a good one must be given.*

**Response**: Thank you for your suggestion. We have revised the conclusion section to provide a more comprehensive and meaningful summary of the study's main findings. Specifically, we summarize the key conclusions of the research, including how fund style drift affects fund performance and how our classification of style drift motivations adds new insights to existing literature discrepancies. Based on this analysis, we also propose policy implications, highlighting how fund companies and regulatory authorities can use the findings to better understand the role of style drift in fund management and make more informed decisions.

*• The implications and recommendations must be related to the results of the study*

**Response**: Thank you for your valuable comment. We have revised the conclusion section to ensure that the implications and recommendations are directly aligned with the research findings. We explicitly connect the results to the implications for fund managers and investors, emphasizing that understanding style drift—especially style drift driven by manager skill—can enhance performance while avoiding trend-chasing drift can prevent negative impacts on returns. Based on our findings, we recommend that fund managers actively manage style drift driven by market opportunities and manager skills, rather than following short-term trends. Additionally, regulatory authorities can use these findings to develop guidelines that balance the benefits and drawbacks of style drift in fund strategies.

*• More cutting-edge limitations and direction for future studies must be stated*

**Response**: Thank you for your suggestion. In the Discussion part, we have added the limitations of the study and directions for future research to better reflect cutting-edge perspectives. This paper uses industry allocation changes to measure style drift, which effectively captures the intensity and frequency of drift, but does not precisely analyze the direction of drift. Future research could build on the existing methods by incorporating measurements of drift direction to more accurately assess the impact of industry performance on funds and analyze fund industry preferences throughout economic cycles. Additionally, this paper does not explore the transmission mechanisms of style drift within the fund network and its impact on the market. The fund market is a highly interconnected network, and the style drift of individual funds may spread to other funds through overlap in holdings and market sentiment, triggering market volatility. Future research could adopt complex network analysis to investigate the propagation of style drift between funds and provide regulatory authorities with early warning systems to identify systemic risks and enhance market stability.
